# Comparison of laparoscopic cholecystectomy in children at paediatric centres and adult centres: a systematic review and meta-analysis

**DOI:** 10.1308/rcsann.2023.0041

**Published:** 2024-03-06

**Authors:** A Sinha, A Mattson, I Njere, CK Sinha

**Affiliations:** ^1^ East and North Hertfordshire NHS Trust, UK; ^2^ St George’s University Hospitals NHS Foundation Trust, UK; ^3^ Royal Devon University Healthcare NHS Foundation Trust, UK

**Keywords:** Laparoscopic cholecystectomy, Paediatric, Paediatric centre, Adult centre, Children

## Abstract

**Introduction:**

Paediatric laparoscopic cholecystectomy (LC) is performed by both paediatric and adult surgeons. The aim of this review was to compare outcomes at paediatric centres (PCs) and adult centres (ACs).

**Methods:**

A literature search was conducted, in accordance with PRISMA (Preferred Reporting Items for Systematic reviews and Meta-Analyses) guidelines, for papers published between January 2000 and December 2020. Statistical analysis was performed using Stata^®^ version 16 (StataCorp, College Station, TX, US).

**Results:**

A total of 92 studies involving 74,852 paediatric LCs met the inclusion criteria. Over half (59%) of the LCs were performed at ACs. No significant differences were noted in the male-to-female ratio, mean age or mean body mass index between PCs and ACs. The main indications were cholelithiasis (34.1% vs 34.4% respectively, *p*=0.83) and biliary dyskinesia (17.0% vs 23.5% respectively, *p*<0.01). There was no significant difference in the median inpatient stay (2.52 vs 2.44 days respectively, *p*=0.89). Bile duct injury was a major complication (0.80% vs 0.37% respectively, *p*<0.01). Reoperation rates (2.37% vs 0.74% respectively, *p*<0.01) and conversion to open surgery (1.97% vs 4.74% respectively, *p*<0.01) were also significantly different. Meta-analysis showed no significant difference in overall complications (*p*=0.92).

**Conclusions:**

The number of LCs performed, intraoperative cholangiography use and conversion rates were higher at ACs whereas bile duct injury and reoperation rates were higher at PCs. Despite a higher incidence of bile duct injury at PCs, the incidence at both PCs and ACs was <1%. In complex cases, a joint operation by both paediatric and adult surgeons might be a better approach to further improve outcomes. Overall, LC was found to be a safe operation with comparable outcomes at PCs and ACs.

## Introduction

Laparoscopic cholecystectomy (LC) is a common procedure in the paediatric population.^1^ Children have lower rates of gallbladder disease than adults but recent studies suggest a rising incidence and an aetiological shift away from haematological disorders.^2,3^ Childhood obesity is overtaking haemolytic disease as the main risk factor in paediatric cholelithiasis.^2^ Along with cholelithiasis, the incidence of biliary dyskinesia has also increased dramatically, leading to a rise in the overall prevalence of gallbladder pathology in children.^1,2,4–6^

Currently, paediatric LC is performed at both paediatric and adult surgical centres. It is often suggested that the difference in the experience of these two settings influences patient outcomes. Paediatric surgeons receive specialised training on handling paediatric tissue, variations in congenital anomalies and techniques specific to surgery in the paediatric population whereas adult surgeons experience larger numbers of cholecystectomies across the adult population. The effect of surgical experience (mostly through operative volume) on outcomes has been studied^7^ but there is limited published evidence on the effect of specialty experience on outcomes. The aim of this systematic review and meta-analysis was to compare the outcomes and experiences of paediatric LC at paediatric centres (PCs) and adult centres (ACs).

## Methods

This study was conducted in accordance with the PRISMA (Preferred Reporting Items for Systematic reviews and Meta-Analyses) guidelines.^8^ A literature search was performed for papers published between January 2000 and December 2020 using the PubMed^®^, Embase^®^, Web of Science™, ScienceDirect^®^ and Cochrane Central Register of Controlled Trials databases. The following Medical Subject Headings or combinations of terms were employed: “laparoscopic”, “cholecystectomy”, “paediatric”, “pediatric” and “child”. Articles identified by the search results as well as from the reference lists of major review articles were reviewed.

### Study selection criteria

Titles and abstracts were screened for relevance before full-text articles were obtained and again screened by two authors (AS and AM). Studies that met the following inclusion criteria were selected: i) written in English; ii) patients aged ≤19 years; and iii) studies involved LC. Papers that did not report outcomes of LC numerically, were not yet published, reported duplicate data, or were unavailable in the full form online or through university library access were excluded.

### Data extraction

Two authors (AS and AM) independently extracted data and checked for discrepancies among papers that met the inclusion criteria. Disagreements were resolved by consensus with the senior authors (IN and CKS). Data extracted included: author names; year of publication; type of study (prospective or retrospective); study size; type of centre at which the LCs were performed (PCs, ACs or both); population demographics; and procedures, complications and outcomes. Data were entered into Excel^®^ (Microsoft, Redmond, WA, US) for analysis.

### Data analysis

Statistical analysis was performed using Stata^®^ version 16 (StataCorp, College Station, TX, US). Medians and means were compared using the Mann–Whitney U test and the unpaired t-test. Data were compared using the chi-squared test with Yates’ correction and Fisher’s exact test. Pooled estimates of outcomes were calculated using a random effects model. The homogeneity of studies was evaluated with the I^2^ statistic and Cochran’s Q test, and assessed visually by means of a funnel plot. Small study effects were analysed with Egger’s regression test. Publication bias was evaluated using non-parametric trim-and-fill analysis. Analysis of the outcomes of LC was stratified according to the type of centre (PCs vs ACs). A *p*-value of <0.05 was deemed statistically significant.

## Results

Overall, 92 papers describing a total of 74,852 patients (31,037 at PCs and 43,815 at ACs) met the inclusion criteria. There were 14 prospective and 78 retrospective studies. Table 1 summarises the studies that met the inclusion criteria. The patient demographics at the PCs and ACs are compared in Table 2. There were no statistically significant differences in the demographics between the two groups.

**Table 1:** Summary of the 92 studies that met the inclusion criteria

Author	Study type	Patients	Centre
Calabro, 2020	Retrospective	31	Paediatric
Dekonenko, 2020	Retrospective	13	Paediatric
Ducey, 2020	Retrospective	101	Paediatric
Esposito, 2020	Prospective	12	Paediatric
Fishman, 2020	Retrospective	67	Paediatric
Pelizzo, 2020	Retrospective	26	Paediatric
Yeh, 2020	Retrospective	250	Adult
Gee, 2019	Retrospective	56	Paediatric
Esposito, 2019	Retrospective	215	Paediatric
Esposito, 2019	Prospective	5	Paediatric
Overman, 2019	Retrospective	81	Paediatric
Pogorelić, 2019	Retrospective	33	Paediatric
Utria, 2019	Retrospective	129	Adult
Akhtar-Danesh, 2018	Retrospective	3,519	Both
Frybova, 2018	Retrospective	147	Paediatric
Krishna, 2018	Retrospective	236	Adult
Nolan, 2018	Retrospective	71	Adult
Noviello, 2018	Retrospective	24	Paediatric
Tom, 2018	Retrospective	8,117	Both
Cairo, 2017	Retrospective	5,046	Paediatric
Graves, 2017	Prospective	11	Paediatric
Pandian, 2017	Retrospective	1,611	Adult
Rosales-Velderrain, 2017	Prospective	14	Paediatric
Seifarth, 2017	Retrospective	98	Paediatric
Gonzalez, 2016	Retrospective	100	Paediatric
Gould, 2016	Prospective	116	Paediatric
Lin, 2016	Retrospective	9	Paediatric
Sacco Casamassima, 2016	Retrospective	2,050	Both
Suh, 2016	Retrospective	19	Adult
Dalton, 2015	Retrospective	227	Paediatric
Farach, 2015	Retrospective	151	Paediatric
Jones, 2015	Retrospective	17	Paediatric
Kim, 2015	Retrospective	24	Paediatric
Mahdi, 2015	Prospective	4	Paediatric
Muller, 2015	Retrospective	36	Paediatric
Seims, 2015	Retrospective	210	Paediatric
Kelley-Quon, 2014	Retrospective	6,863	Both
Langballe, 2014	Retrospective	186	Both
Ruparel, 2014	Retrospective	5	Paediatric
Zeidan, 2014	Retrospective	202	Paediatric
Bibza, 2013	Retrospective	95	Paediatric
Jawaheer, 2013	Prospective	25	Paediatric
Karakuş, 2013	Retrospective	27	Paediatric
Leinwand, 2013	Prospective	18	Paediatric
Millan, 2013	Retrospective	29	Paediatric
Short, 2013	Retrospective	42	Paediatric
Al-Salem, 2012	Retrospective	59	Paediatric
Chen, 2012	Retrospective	3,596	Both
Chowdhary, 2012	Prospective	21	Paediatric
Knott, 2012	Retrospective	41	Paediatric
Misauno, 2012	Prospective	7	Paediatric
Tannuri, 2012	Retrospective	204	Paediatric
Chandler, 2011	Retrospective	69	Paediatric
Emami, 2011	Retrospective	25	Paediatric
Garcia-Henriquez, 2011	Retrospective	54	Paediatric
Garey, 2011	Retrospective	24	Paediatric
Germani, 2011	Retrospective	7	Paediatric
Liuming, 2011	Retrospective	39	Paediatric
Lyons, 2011	Retrospective	37	Paediatric
Menon, 2011	Retrospective	102	Paediatric
Mesas Burgos, 2011	Retrospective	10	Paediatric
Raval, 2011	Retrospective	28,243	Both
Garey, 2010	Retrospective	312	Paediatric
Turial, 2010	Prospective	22	Paediatric
Alwabari, 2009	Retrospective	47	Paediatric
Esposito, 2009	Retrospective	100	Paediatric
Chan, 2008	Prospective	27	Paediatric
Della Corte, 2008	Retrospective	50	Paediatric
Goers, 2008	Retrospective	79	Paediatric
Haricharan, 2008	Retrospective	23	Paediatric
Siddiqui, 2008	Retrospective	184	Paediatric
St Peter, 2008	Retrospective	224	Paediatric
Currò, 2007	Retrospective	26	Paediatric
Issa, 2007	Retrospective	40	Paediatric
Al-Mulhim, 2006	Retrospective	54	Adult
Balaguer, 2006	Retrospective	9,931	Paediatric
Bonnard, 2005	Retrospective	12	Paediatric
Karplus, 2005	Retrospective	65	Paediatric
Seleem, 2005	Prospective	12	Adult
Campbell, 2004	Retrospective	16	Paediatric
Mah, 2004	Retrospective	48	Paediatric
Suell, 2004	Retrospective	54	Paediatric
Al-Homaidhi, 2002	Retrospective	10	Paediatric
Sakopoulos, 2002	Retrospective	5	Paediatric
Sandoval, 2002	Retrospective	10	Paediatric
Esposito, 2001	Retrospective	110	Paediatric
Mattioli, 2001	Prospective	58	Paediatric
Michail, 2001	Retrospective	48	Paediatric
Séguier-Lipszyc, 2001	Retrospective	29	Adult
Shah, 2001	Retrospective	5	Paediatric
Ure, 2001	Retrospective	8	Paediatric
Waldhausen, 2001	Retrospective	100	Paediatric

**Table 2:** Patient demographics by type of centre

	Paediatric centres			Adult centres			*p*-value
	Value	Studies	Patients	Value	Studies	Patients	
Total cases			31,037			43,815	
Male-to-female ratio	1:2.2	56	18,832	1:2.7	10	2,418	0.60
Mean age (years)	12.5	66	9,168	12.1	10	2,418	0.68
Mean weight (kg)	49.9	20	1,638	52.2	3	102	0.85
Mean body mass index (kg/m^2^)	24.9	16	1,170	22.7	4	634	0.32

### Predisposing risk factors

Thirty-one studies from PCs and six from ACs reported on haematological disorders. A history of haematological disorders was significantly more common in LCs performed at ACs than at PCs (18.7% vs 15.8%, *p*<0.01).

### Indications

Forty-six papers from PCs and five from ACs highlighted cholelithiasis as the main indication (34.1% vs 34.4% respectively, *p*=0.83) (Table 3). Biliary dyskinesia was reported in 26 studies from PCs and 4 from ACs, and this was the second most common indication in both groups (17.0% vs 23.5% respectively, *p*<0.01).

**Table 3:** Indications and presentations for laparoscopic cholecystectomy by type of centre

	Paediatric centres			Adult centres			*p*-value
	%	Studies	Patients	%	Studies	Patients	
*Indication*							
Cholelithiasis	34.1%	46	8,310	34.4%	5	1,963	0.83
Biliary dyskinesia	17.0%	26	7,221	23.5%	4	2,315	**<0.01**
Haematological disorders	15.8%	31	17,238	18.7%	6	2,513	**<0.01**
Choledocholithiasis	14.9%	28	11,844				
Trauma	1.0%	3	301				
*Presentation*							
Cholecystitis (not specified)	20.1%	37	17,659	40.0%	3	1,880	**<0.01**
Acute cholecystitis	3.4%	35	2,448	11.8%	3	1,915	**<0.01**
Chronic cholecystitis	17.5%	34	2,347	29.3%	3	1,915	**<0.01**
Pancreatitis	7.1%	24	16,819				
Cholangitis	1.1%	4	10,459				
Polyps	2.4%	6	551				

### Presentation

LCs performed at ACs had a significantly higher proportion of presentations of non-specific, acute and chronic cholecystitis (Table 3). Non-specific cholecystitis was mentioned in 37 PC papers with a rate of 20.1% and in 3 AC papers with a rate of 40.0% (*p*<0.01). Acute cholecystitis was reported in 35 PC and 3 AC studies (3.4% vs 11.8% respectively, *p*<0.01) while chronic cholecystitis was noted in 34 PC and 3 AC papers (17.5% vs 29.3%, *p*<0.01).

### Operative metrics

Operative time was mentioned in 11 PC and 2 AC studies. There was no significant difference between median operative times (99 vs 84 minutes respectively, *p*=0.40). Inpatient stay was reported in 50 PC and 9 AC papers. There was no difference in median inpatient stay (2.52 vs 2.44 days respectively, *p*=0.89). Intraoperative cholangiography (IOC) was discussed in 32 PC and 3 ACs studies. IOC was performed more frequently at ACs than at PCs (28.8% vs 25.6%, *p*<0.01). Preoperative endoscopic retrograde cholangiopancreatography (ERCP) was mentioned in 22 PC papers and 1 AC paper. No statistically significant difference in ERCP use was noted (12.1% vs 8.3% respectively, *p*=0.69). The operative metrics are summarised in Table 4.

**Table 4:** Operative metrics, outcomes and management of complications by type of centre

	Paediatric centres			Adult centres			*p*-value
	Value	Studies	Patients	Value	Studies	Patients	
*Operative metrics*							
Median operative time (minutes)	99	11	417	84	2	83	0.40
Median inpatient stay (days)	2.52	50	18,378	2.44	9	2,182	0.89
Intraoperative cholangiography	25.6%	32	17,438	28.8%	3	7,196	**<0.01**
Endoscopic retrograde cholangiopancreatography	12.1%	22	11,713	8.3%	1	12	0.69
Common bile duct exploration	2.6%	9	11,022				
*Outcomes*							
Median overall complication rate	2.60%			2.28%			0.053
Minor complications	1.64%	36	8,898	3.00%	5	9,356	**<0.01**
Bleeding	1.97%	16	1,623	1.60%	1	187	1.0
Bile duct injury	0.80%	6	5,856	0.37%	1	28,243	**<0.01**
Acute chest syndrome	5.50%	5	200	1.60%	1	187	0.056
Chest infection	12.82%	1	39	5.56%	1	54	0.27
Retained stone	1.93%	15	1,453				
Bile leak	2.38%	8	881				
Duodenal perforation	3.70%	5	567				
Clip dislocation	1.08%	3	371				
Intraperitoneal infection	0.88%	2	5,248				
Death	0.20%	1	9,931				
*Management of complications*							
Transfusion	2.42%	8	621	19.81%	3	212	**<0.01**
Reoperation rate	2.37%	5	464	0.74%	1	1,611	**<0.01**
Conversion to open surgery	1.97%	20	12,122	4.74%	3	190	**<0.01**

### Complications

There was no significant difference in overall complication rates between PCs and ACs (2.60% vs 2.28% respectively, *p*=0.053) (Table 4). In terms of major complications, 16 PC studies and 1 AC study noted bleeding, with no significant difference between the two groups (1.97% vs 1.60% respectively, *p*=1.0). Bile duct injury (BDI) was reported in six PC papers and one AC paper. The incidence was found to be significantly higher in PCs than in ACs (0.80% vs 0.37%, *p*<0.01). Acute chest syndrome was discussed in five PC studies and one AC study with rates of 5.50% and 1.60% respectively (*p*=0.056). Chest infection was reported in one PC and one AC paper (12.82% vs 5.56% respectively, *p*=0.27).

A number of other complications were mentioned in papers that reported solely on paediatric LC (Table 4). These included retained stones (with an incidence of 1.93%), bile leak (2.38%), duodenal perforation (3.70%), clip dislocation (1.08%) and intraperitoneal infection (0.88%).

One PC paper reported patient death. This study included 9,931 patients and had a mortality rate of 0.20%. None of the AC papers mentioned patient mortality.

Postoperative transfusion was discussed in eight PC and three AC studies. A rate of 2.42% was found at PCs and 19.81% at ACs (*p*<0.01). Five PC papers and one AC paper reported reoperation. Reoperations were more than twice as common at PCs compared with ACs (2.37% vs 0.74%, *p*<0.01). Twenty PC and three AC studies mentioned the conversion rate of LC to open surgery, with a significantly higher incidence noted in ACs (1.97% vs 4.74%, *p*<0.01) (Table 4).

Minor complications (pain, nausea, fever or port site infection) were reported in 36 PC and 5 AC studies. Rates were almost twice as high in ACs (1.64% vs 3.00%, *p*<0.01; Table 4).

### Meta-analysis

**Figure 1:**
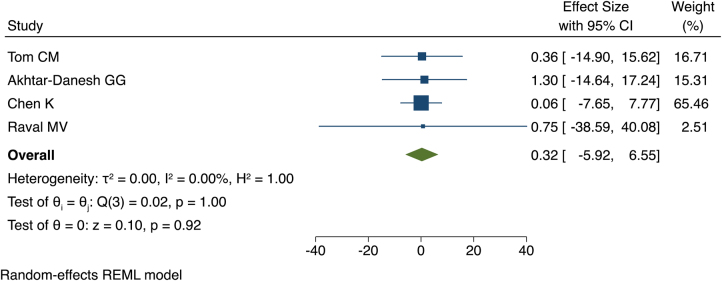
Forest plot showing complication rates of adult versus paediatric centres for the four studies included in the meta-analysis^7,9–11^

**Figure 2:**
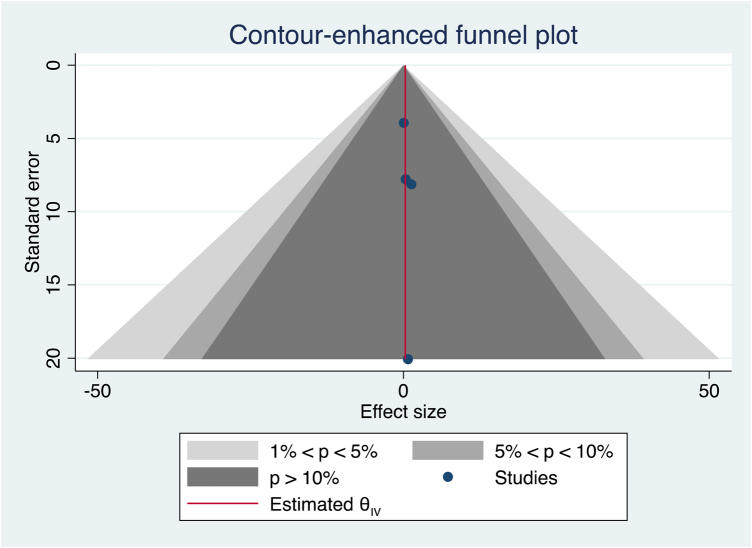
Funnel plot showing homogeneity of the four studies included in the meta-analysis^7,9–11^

Four papers lent themselves to meta-analysis, with no significant difference in complication rates at PCs and ACs (effect size [θ] = 0.316, 95% confidence interval [CI]: −5.921 to 6.553, *p*=0.92) (Figure 1).^7,9–11^ The I^2^ statistic and Cochran’s Q test indicated homogeneity (Figure 1). Homogeneity was assessed visually using a funnel plot, shown in Figure 2. The regression-based Egger test for small study effects indicated no influence of small studies (*p*=0.90). The non-parametric trim-and-fill analysis of publication bias showed no evidence of publication bias (Observed = 0.316, 95% CI: -5.921 to 6.553; Observed + Imputed = 0.316, 95% CI: -5.921 to 6.553).

## Discussion

LC is recognised as the gold standard treatment for symptomatic cholelithiasis. Guidance from the National Institute for Health and Care Excellence for diagnosing and managing gallstone disease in adults is often employed for the paediatric population.^6,12^ This may be owing to the paucity of paediatric data in the literature. Outcomes of paediatric LC have gained more clinical importance with the increasing prevalence of gallbladder disease in this population.^2,3^ Previous work has been undertaken on the outcomes of paediatric versus adult surgeons for inguinal hernia repairs, appendicectomies and surgery for pyloric stenosis.^13–15^ As paediatric surgeons perform smaller volumes of LCs than adult surgeons, an assessment of the specialty effect in cases of LC would be of interest when determining future management approaches for LC in the paediatric population. To our knowledge, this is the largest study comparing the outcomes of paediatric LC between PCs and ACs published so far.

The demographics of populations from both types of centre were comparable. There were no significant differences in sex, age, weight or body mass index.

The main indications for LC in our review were cholelithiasis and biliary dyskinesia, in agreement with other studies.^10,16^ Cholelithiasis was the most common indication for LC in both AC and PC groups, as also noted by previous authors.^3–6^ The incidence of cholelithiasis was found to be 34.1% in PCs and 34.4% in ACs, which underscores its continued high prevalence as similar findings have been reported by other authors during a similar period.^2,4–6^ The growing prevalence of cholelithiasis among the paediatric population may be attributed to lifestyle factors increasing risk but also to more widespread availability and use of diagnostic facilities such as ultrasonography. The main predisposing factors for cholelithiasis in children are haematological disorders and obesity. Obesity is progressively replacing haematological disorders as the main risk factor for cholelithiasis, perhaps owing to diet and exercise factors influencing children’s health today.^1,2,4–6^

Biliary dyskinesia is gaining prominence as an indication for cholecystectomy. It has been reported as constituting 10–40% of the indications for gallbladder removal in children, with several studies observing a rise in incidence.^17–21^ This is mainly in papers from the US. Biliary dyskinesia as an indication for cholecystectomy has been dogged by inconsistent diagnostic criteria and controversy.^22,23^ In this review, the rate of biliary dyskinesia as an indication for LC in ACs was significantly higher than in PCs. The rates of 17.0% in PCs and 23.5% in ACs highlight the different approaches to biliary dyskinesia by the two groups.

Our study identified that haematological disorders were risk factors in 15.8% of LC cases at PCs compared with 18.7% at ACs, with previously reported figures ranging from 10% to 25%.^19,24^ This difference may be due to paediatric surgeons treating stones related to haematological disorders largely with expectant management rather than with LC.^25^

Cholecystitis presents less commonly in children than in adults.^17^ As a result, management guidelines for adults are often employed, with the specific paediatric experience being underreported.^17^ Our rates of cholecystitis leading to LC in 20.1% of cases in PCs and 40.0% in ACs agree with the existing literature, and confirm that it is the main mode of presentation of gallstones in children.^17,26^

Cholecystectomy is often used to treat “hot gallbladders” (during presentation of acute cholecystitis) in adults.^27,28^ Indeed, a large prospective study identified a third of their cases as acute cholecystitis before proceeding to LC.^26^ Specifically in our review, acute cholecystitis led to LC for 3.4% of the cases at PCs whereas this was 11.8% at ACs. This may reflect adult practice being applied to the paediatric population, with a greater proportion of paediatric “hot gallbladders” being operated on. The propensity for PCs to manage a larger proportion of these presentations conservatively may also contribute to this difference.

The exact incidence of choledocholithiasis in the paediatric population is largely unknown. It is reported to be 10% in adults.^29^ The incidence in children undergoing cholecystectomy has been found to range between 9.7% and 11% whereas in adults undergoing cholecystectomy, it is reported to be 5–18%.^17,19^ Our study's incidence of 14.9% in children undergoing LC in PCs is higher than previous reports for paediatric populations, and may suggest that paediatric rates are approaching those for adults with the increasing prevalence of cholelithiasis risk factors and presentations.

Literature on the incidence of gallstone pancreatitis in children is limited. Our rate of 7.1% in PCs among children undergoing LC is supported by the existing literature, which reports a range of 3–8% in the paediatric population.^17,19^

Our review found operative metrics to be comparable across PCs and ACs. There was no significant difference between median operative time, median inpatient stay or preoperative ERCP use. Common bile duct exploration was performed in 2.6% of cases at PCs, with no comparable data available from ACs. Interestingly, IOC use was significantly more frequent at ACs than at PCs (28.8% vs 25.6% respectively, *p*<0.01). This is in agreement with previous reports of IOC in paediatric LC ranging from 17% to 39%.^17,30^

IOC represents a valuable tool in delineating biliary anatomy, identifying common bile duct stones and confirming BDI. The increased use of IOC in ACs could be due to adult surgeons being more comfortable with bile duct explorations, which could follow IOC, as some argue that regular IOC use upskills the operating team for when intervention is required.^31^ Conversely, others argue that routine use increases the risk for iatrogenic injury and radiation exposure, and that IOC is no more sensitive to choledocholithiasis than preoperative magnetic resonance imaging.^31^ However, the fact that only limited data are available makes reaching conclusions difficult. Indeed, this is also shown through the lack of consistent guidance on when IOC should be used.^32^

BDI is one of the most serious complications of LC. The risk quoted in the adult literature ranges from 0.02% to 0.77%.^11,30,33–36^ Our review has shown a significant difference in this complication rate between PCs and ACs (0.80% vs 0.37% respectively, *p*<0.01). The reason for this difference is likely to be multifactorial. Contributing factors may include higher operative volumes leading to more experience at ACs, less frequent use of IOC at PCs leading to more frequent misidentification of biliary anatomy predisposing to BDI, and PCs possibly representing specialist referral hubs for patients with complex presentations and anatomy or who are being referred to a PC subsequent to a complication occurring elsewhere.^11,30,31,33,37,38^

Confounding variables are also possible and the difference may not be a direct consequence of the differential IOC use. Greater IOC use could suggest a generally safer surgical technique or greater experience on the surgeon’s part. Additionally, if PCs and ACs have different policies on discharge, this may result in BDI diagnosis after discharge in one group of patients, meaning it may not be captured in the same data as the operation. Other variables such as family centred care near the home, trainee involvement and patients travelling further to a specialist PC could also affect inpatient stay and outcomes.

Raval *et al* gave an overall BDI rate of 0.44% and showed a significantly lower rate at ACs than at PCs.^11^ They also demonstrated significantly higher rates of BDI in patients aged <6 years after controlling for patient, hospital and regional factors as well as disease specific risk factors. They found that PCs had a higher proportion of younger patients (<6 years). This differs from our findings as there was no significant difference in patient demographics but these data were not reported in all studies and were more limited from ACs than from PCs.

It is important to mention this while consenting these patients and an offer to transfer these children to an AC may be discussed with patients whose age or weight approaches that of the adult population. Nevertheless, further work should be undertaken to characterise the difference in BDI rates to clarify the advice we provide to patients, and other complications and its practical feasibility need to be considered in decision making. However, the figures we found at both types of centre are low (<1%), in line with findings from the existing literature. This suggests that LC is a safe procedure at both PCs and ACs.

The reoperation rate was found to be higher at PCs than at ACs (2.37% vs 0.74%) but conversion to open surgery showed the opposite trend, being higher at ACs than at PCs (4.74% vs 1.97%). It is possible that the higher reoperation rate at PCs is due to less frequent IOC use missing BDI, abnormal anatomy leading to trauma/bleeding and common bile duct stones requiring further operations. The higher conversion rate at ACs may represent the higher rates of “hot gallbladders” that are operated on as multiple studies have shown an association with acute inflammation and increased incidence of conversion to open surgery.^26,39,40^

Death was reported in only one study, involving nearly 10,000 children.^41^ The mortality rate was 0.20%. This is an interesting finding and comparable with mortality statistics for cholecystectomy (laparoscopic and open) from adult studies (0.1–0.7%).^42^

Previous studies have demonstrated the importance of surgical volume on patient outcomes.^7,14,15^ In many of these cases, it is more important than surgical specialty. For example, Kelley-Quon *et al* show significantly higher complication rates in low volume centres and surgeons.^15^ It is understandable that surgical volume will be one of the most important factors affecting outcomes and given the epidemiology of the disease, this can lead to a higher number of operative cases in the hands of adult surgeons. However, we wanted to look at specialty differences, and the differences in training, technique, facilities and multidisciplinary team care that may inform referral and operative choices for paediatric patients in future. There is considerable work demonstrating the utility of subspecialty specific surgical training leading to better outcomes in oncological, vascular and paediatric surgery.^43–50^

Some studies also discuss findings and variables that were outside of the scope of this review but that may warrant further investigation in future.^11,38^ Raval *et al* reported rates of BDI in non-White populations that were 49% higher than for White patients and elective patients were twice as likely to suffer BDI as patients undergoing emergent surgery.^11^ These factors may be contributing to the difference in our BDI rates and should be considered in data collection for future work. Alternatively, they may indicate patient groups where higher intraoperative vigilance is required. Additional control variables would allow for better comparison of specialty dependent outcomes in future. Advances in paediatric surgery can also be characterised and their outcomes compared, such as the use of single port LC in children.^51^

### Study limitations

Our meta-analysis contained only retrospective studies^7,9–11^ and many of the papers included in our review described single centre studies, with incomplete and non-randomised data from both PCs and ACs. Heterogeneity affected individual study demographics, methods, definitions, results and interpretations. Minor complications (such as postoperative pain and nausea) may have been subject to a reporting bias as these could potentially have been overlooked by surgeons disregarding them as complications. The limited number of papers with high grade evidence was another important limitation of this study and long-term outcomes were not regularly collected in many papers. Nevertheless, the strength of this review lies in the provision of the largest dataset to date looking at paediatric LCs, and the important comparison of outcomes and experience from PCs and ACs.

## Conclusions

There was no significant difference in the demographics or overall complications for paediatric LC between PCs and ACs. The number of LCs performed, IOC use and conversion rates were higher at ACs whereas BDI and reoperation rates were higher at PCs. Even though a higher incidence of BDI was seen in PCs, the incidence at both PCs and ACs was below 1%, which was reassuring. In complex LC cases, a joint operation by both paediatric and adult surgeons might be a better approach to further improve outcomes. Overall, LC was found to be a safe operation with comparable outcomes at both PCs and ACs.
